# Case Report: Neratinib Therapy Improves Glycemic Control in a Patient With Type 2 Diabetes and Breast Cancer

**DOI:** 10.3389/fendo.2022.830097

**Published:** 2022-03-17

**Authors:** Vasileios Angelis, Stephen R. D. Johnston, Amin Ardestani, Kathrin Maedler

**Affiliations:** ^1^The Royal Marsden NHS Foundation Trust & The Institute of Cancer Research, London, United Kingdom; ^2^Centre for Biomolecular Interactions Bremen, University of Bremen, Bremen, Germany; ^3^Department of Molecular Medicine, School of Advanced Technologies in Medicine, Tehran University of Medical Sciences, Tehran, Iran

**Keywords:** diabetes, neratinib, MST1, EGFR, HER2, breast cancer, comorbidity

## Abstract

A critical decline of functional insulin-producing pancreatic β-cells is the central pathologic element of both type 1 and type 2 diabetes. Mammalian Sterile 20-like kinase 1 (MST1) is a key mediator of β-cell failure and the identification of neratinib as MST1 inhibitor with potent effects on β-cell survival represents a promising approach for causative diabetes therapy. Here we report a case of robust glycemia and HbA1c normalization in a patient with breast cancer-T2D comorbidity under neratinib, a potent triple kinase inhibitor of HER2/EGFR and MST1. The patient, aged 62 years, was enrolled in the plasmaMATCH clinical trial and received 240 mg neratinib once daily. Neratinib therapy correlated with great improvement in glucose and HbA1c both to physiological levels during the whole treatment period (average reduction of random glucose from 13.6 ± 0.4 to 6.3 ± 0.5 mmol/l and of HbA1c from 82.2 ± 3.9 to 45.6 ± 4.2 mmol/mol before and during neratinib). 18 months later, when neratinib was withdrawn, random glucose rapidly raised together with high blood glucose fluctuations, which reflected in elevated HbA1c levels. This clinical case reports the combination of HER2/EGFR/MST1-inhibition by neratinib for the pharmacological intervention to effectively restore normoglycemia in a patient with poorly controlled T2D and suggests neratinib as potent therapeutic regimen for the cancer-diabetes comorbidity.

**Graphical Abstract:**
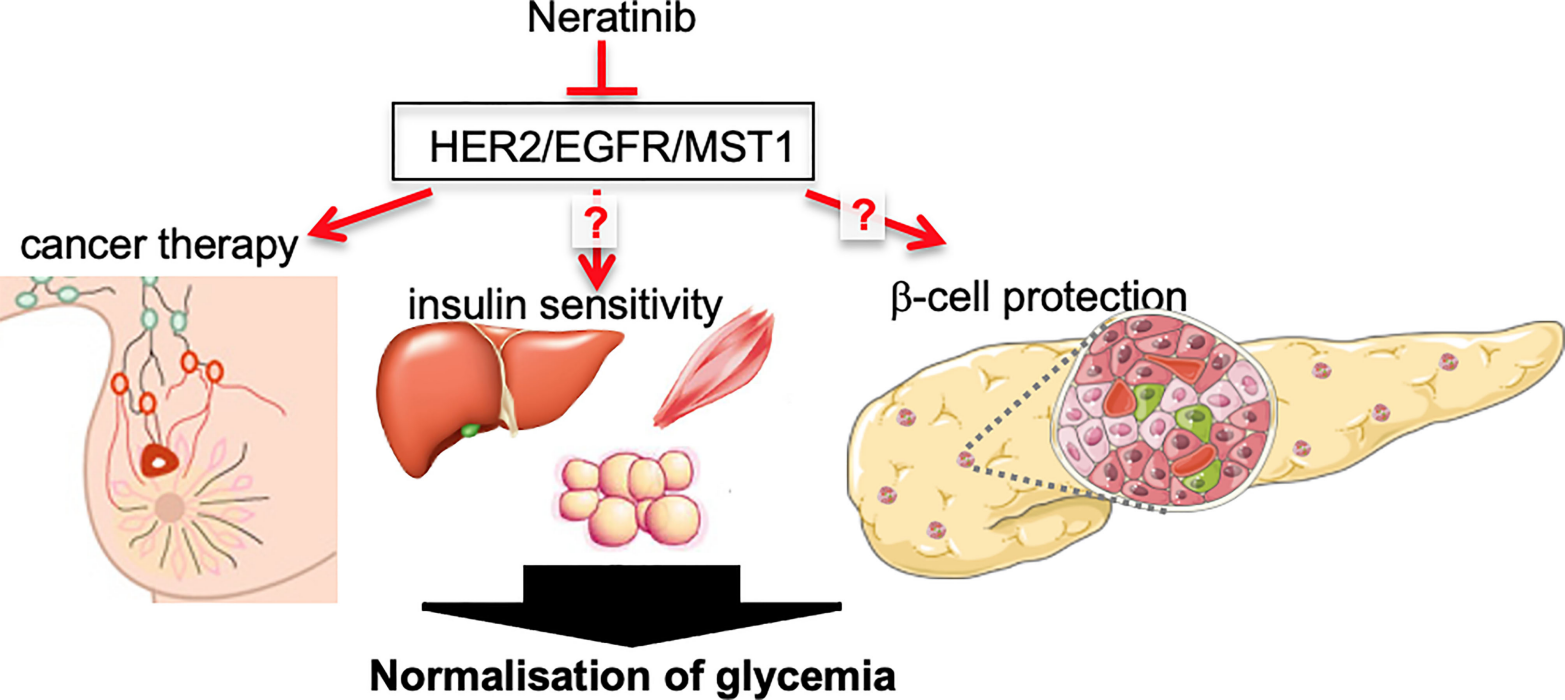
Angelis et al. describe a case report of robust glycemia and HbA1c normalization in a patient with breast cancer-T2D comorbidity under neratinib therapy, a potent triple kinase inhibitor of HER2/EGFR and MST1, which may restore glycemia through both improving insulin sensitivity and β-cell survival. (Figure created using smart servier medical art under https://creativecommons.org/licenses/by/3.0/).

## Introduction

Overexpression of the tyrosine kinase human epidermal growth factor receptor 2 (HER2, also named ErbB-2) is seen in 25-30% of breast cancer patients and is associated with poor outcome in patients with primary disease (1). Blocking HER2 by neratinib is highly active against HER2-overexpressing human breast cancer; it reduces HER2 receptor autophosphorylation and functions as an irreversible inhibitor (2). Neratinib was approved by the FDA as an extended adjuvant therapy in early HER2+ breast cancer based on the ExteNET study (3).

Aging, obesity, western diet and sedentary life style are well-known causes of the severe co-morbidity of cancer and diabetes (4). Therapeutic targeting of epidermal growth factor receptor (EGFR, also named ErbB-1/HER1)/HER2 signaling, a classical oncogenic pathway, which is immediately connected to metabolic disease, has a high potential for controlling the cancer-diabetes co-morbidity. Two independent reports show profound reduction of fasting glucose levels and normalization of HbA1c in two lung cancer patients with severe T2D treated with the EGFR inhibitor erlotinib (5, 6). Importantly, HER2-positive cancers show enhanced glycolysis, together with metabolic stress-response (7), which provides a direct link from HER2 and cancer to metabolic derangement. A large population-based cohort study correlates high levels of HER2 significantly with a higher risk of diabetes (8), and improvement of insulin sensitivity by metformin not only masters glycemia, but also downregulates HER2 expression (9). In support of the recent discovery of neratinib as additional inhibitor of Mammalian Sterile 20-like kinase 1 (MST1, also known as STK4), which is a ubiquitously expressed serine/threonine kinase and a major core kinase in the Hippo signaling pathway, an important observation in pancreas cancer management was that neratinib reduces K-RAS hyperactivity, the common cancer oncogenic mark, through inhibition of MST1/3/4 and Hippo signaling (10). All these previous studies make neratinib a potent option for the therapy of diabetes-cancer comorbidity.

A critical decline of functional insulin-producing pancreatic β-cells represents a central pathologic element of both type 1 and type 2 diabetes (T1D/T2D). MST1 is a principal contributor of β-cell death and dysfunction. Abnormal activation of MST1 under diabetogenic conditions correlates with β-cell loss, impaired insulin secretion and degradation of pancreatic and duodenal homeobox 1 (PDX1), a β-cell transcription factor highly important for β-cell identity, survival and function (11). Aberrant MST1 activity is not unique to β-cells in diabetes; MST1 hyper-activation contributes to diabetic complications as cardiomyopathy, nephropathy and cardiovascular disease (12). Genetic MST1 deficiency markedly restores β-cell function and survival and leads to protection of β-cell mass and normoglycemia in mouse models of diabetes (11). Through a biochemical MST1 inhibition screen across drug-like kinase inhibitors, neratinib (initially named HKI-272), the dual inhibitor of EGFR/HER2 was identified as potent MST1 inhibitor and improves glycemia and restores β-cell mass and survival in rodent diabetes models as well as in stressed isolated human islets (13). Half maximal inhibitory concentration (IC50) of neratinib determined against purified EGFR and MST1 were at a nanomolar range (EGFR IC_50_ = 1.79nM; MST1 IC_50_ = 37.7nM) (13); IC50 values for EGFR and Her2 were comparable in similar assays (EGFR IC_50_ = 10.8 and HER2 IC_50_ = 9.2 nM) (14).

Here we report a case of robust glycemia normalization in a patient with breast cancer-T2D comorbidity under neratinib therapy.

## Case Report

A 62-year-old Caucasian woman with a previous history of triple negative breast cancer, for which she underwent a wide local excision and adjuvant radiotherapy 6 years ago, presented with erythema and swelling in the right breast. She had a medical history of T2D, hypertension, ischemic heart disease with a previous myocardial infarction that required angioplasty, paroxysmal atrial fibrillation and also a history of rheumatic fever in childhood. Her diabetes was controlled by the combination of an oral anti-hyperglycemic regime of metformin (1g twice daily), gliclazide (160 mg twice daily) and sitagliptin (100 mg once daily; **Table 1**), other medications are irbesartan, indapamide, diltiazem, aspirin and pravastatin.

**Table 1:** Lines of systemic anticancer and diabetes therapy received.

Breast cancer treatments and duration	Cancer regime	Corticosteroids	Diabetes regime
1^st^ Line (January 2017 – July 2017)	Capecitabine	none	metformin 1 g/2x/d sitagliptin 100 mg 1xd gliclazide 160 mg 2xd
2^nd^ line (July 2017 – January 2018)	Paclitaxel	dexamethasone 4 mg i.v. 1x/week	metformin 1 g/2x/d sitagliptin 100 mg 1x/d gliclazide 160 mg 2x/d
3^rd^ line (March 2018 – August 2018)	Neratinib 240 mg 1x/d	none	metformin 1 g/2x/d sitagliptin 100 mg 1xd gliclazide 160 mg 2xd
(September 2018 – September 2019)			metformin 1 g/1x/d, 500 mg/1x/d sitagliptin 100 mg 1xd gliclazide 160 mg 2xd
4^th^ line (October 2019- September 2020)	Docetaxel Trastuzumab Pertuzumab	dexamethasone 8 mg i.v. 2x/d for 3 days every 3 weeks	metformin 1 g/1x/d, 500 mg/1xd sitagliptin 100 mg 1x/d gliclazide 160 mg 2x/d

A breast biopsy confirmed the presence of poorly differentiated metastatic breast carcinoma invading the dermis, with axillary lymph node involvement and systemic oral anti-cancer therapy with capecitabine was commenced. This regime offered only some benefit to the patient for 6 months, after which further progression in the breast and axilla warranted the change of therapy to intravenous paclitaxel, to which she became resistant after 6 months of therapy, which again required a change of treatment. As the patient had a good performance status, she was recruited in the plasmaMATCH multicohort, open-label, non-randomized, phase 2a clinical trial, that aimed to assess the accuracy of circulating tumor DNA (ctDNA) in advanced breast cancer to select patients for mutation-directed specific therapy (15). CtDNA screening for a selected number of clinically relevant genetic alterations confirmed the presence of a HER2 mutation. As a result, she was commenced on neratinib. The patient reported symptoms of grade 1 diarrhea, the most common neratinib side effect, which was well controlled with loperamide. Other side effects were grade 1 nausea, gastro-esophageal reflux symptoms as well as nail changes, all which were controlled by supportive loperamide anti-sickness and topical medications at the beginning of neratinib therapy and thereafter well tolerated by the patient.

**Figure 1:**
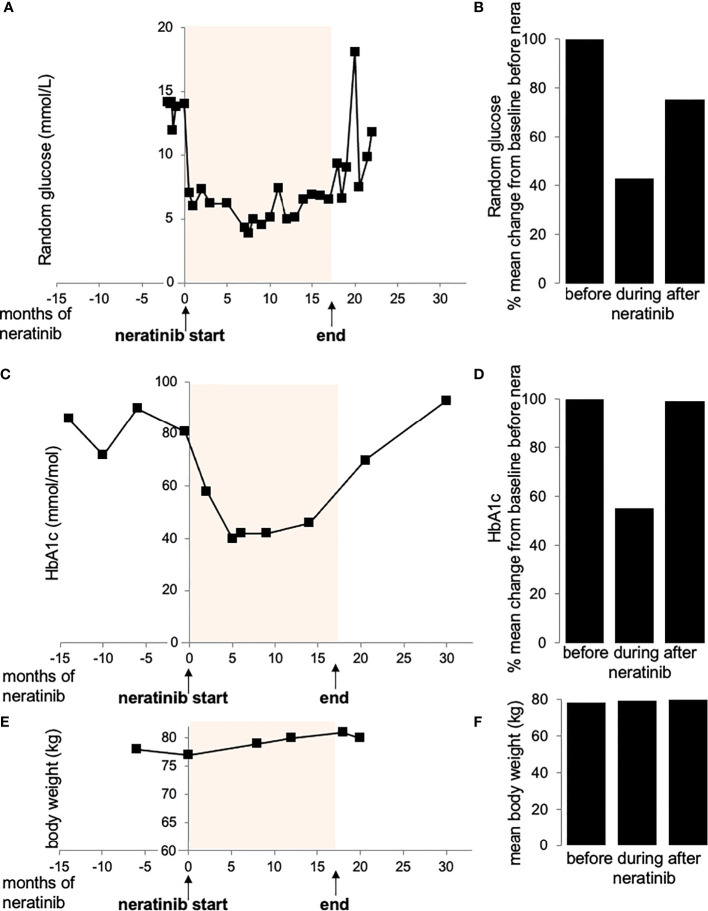
Random glucose **(A, B)**, HbA1c **(C, D)** and body weight **(E, F)** values before, during and after treatment with neratinib. (Figure created using smart servier medical art under https://creativecommons.org/licenses/by/3.0/)

Glycemia and HbA1c was controlled throughout the whole observation period before, during and after neratinib therapy. Soon after commencing neratinib, the patient noticed great improvement in glucose measurements with an average 57% reduction of random glucose to physiological levels (from 13.6 ± 0.4 mmol/L before to 6.3 ± 0.5 mmol/L after neratinib, **Figures 1A, B**). The improvement in glycemia was also reflected by an average 44% reduction of glycated hemoglobin (HbA1c; reduction from 82.2 ± 3.9 mmol/mol before to 45.6 ± 4.2 mmol/mol after neratinib, **Figures 1C, D**). Such physiological glycemic control was maintained throughout her neratinib therapy. In response, her diabetes medication was adjusted and metformin reduced (**Table 1**). 18 months later, the patient reported new symptoms of neck and axillary discomfort. Mammography, ultrasonography and breast magnetic resonance imaging and a core biopsy indicated the presence of a HER2 amplified breast cancer, which required a change in the systemic anti-cancer therapy to docetaxel combined with anti-HER2 antibodies trastuzumab and pertuzumab. The discontinuation of neratinib rapidly raised random glucose; we observed a poor glycemic control with high fluctuation in blood glucose, which was followed by an increase in HbA1c 10 months later back to levels before neratinib therapy (**Figure 1**). The change in the therapeutic regime to docetaxel combination therapy included steroids initially to prevent a possible hypersensitivity reaction and reduce initial nausea, the short duration of 3 days of dexamethasone during actual chemotherapy (8 mg dexamethasone twice daily over three days with three-week intervals; **Table 1**) used in this setting is unlikely to lead to chronic steroid-induced hyperglycemia, and would especially normalize during the 3 weeks of steroid break. Patient’s body weight remained stable with only rare fluctuations through the treatment and the whole observation period (BW=79.2 kg ±0.6; **Figures 1E, F**).

In summary, we have observed robust glycemic control in a patient with initially poorly controlled T2D during the 18-month neratinib therapy in a phase 2a clinical trial against HER2 mutation associated breast cancer. The full normalization of HbA1c highly improved the patient’s life and her psychological status, despite the initially experienced neratinib side effect of mild grade 1 diarrhea, which could be rapidly controlled.

## Discussion

From previous studies in human islets and diabetic mouse models, we observed restoration of β-cell mass and survival to a physiological level under neratinib. Thus, we had expected a long-term improvement after the wash out-period. However, this study shows that glycemia fluctuations have occurred relatively rapidly at one month after neratinib discontinuation. As we have no estimates on the levels of β-cell function before, during and after neratinib therapy, such should be investigated in a follow-up study by including metabolic parameters as well as pancreas MRI/Pet-Scan studies, in order to assess β-cell protection *in vivo* as seen before in human islets *in vitro* and in mouse models. Insulin and C-peptide levels would perfectly help to understand the mechanisms of the observed neratinib-mediated glycemia improvement in this study. Unfortunately, according to the trial restrictions, we had no opportunity to access the patient’s serum.

As the patient has been suffering from barely controlled diabetes for many years before her cancer diagnosis, it is possible that the add-on dexamethasone therapy, which was included prior as well as after neratinib, contributed to the severe hyperglycemia experienced by the patient. Chronic corticoids of ≥8 mg/d dexamethasone (or equivalents of approximately 50 mg prednisone or 210 mg hydrocortisone) are well-known inducers of chronic and persistent metabolic derangements progressing to glucocorticoid-induced diabetes with hyperglycemia and insulin resistance (16). However, several clinical examples show that also acute dexamethasone of 8 mg/day over two days was enough to induce insulin resistance in healthy subjects (17), 8 mg dexamethasone once weekly to increase hyperglycemia for up to three days in patients with diabetes in the oncologic setting (18) and even a single dose of 4 mg dexamethasone to increase hyperglycemia during a 24h observation period after surgery, with 8-10 mg further potentiating hyperglycemia (19). Such short-term dexamethasone has been observed to cause a steady state of hyperglycemia within 3h of therapy but returns to baseline after 3 days (18), and also corticoid-induced insulin resistance has been reported to be transient (17). While chronic glucocorticosteroids cause diabetes, acute and intermittent dexamethasone should show recovery during periods when dexamethasone is not administered.

Prior to neratinib, our patient received 4 mg dexamethasone once weekly to prevent hypersensitivity to chemotherapy, which was unlikely to cause chronically high long-term blood glucose/HbA1c levels. Notably, our patient suffered from persistent hyperglycemia and has never returned to baseline during intermittent glucocorticoid therapy. Neratinib fully normalized glycemia for the first time after her diabetes diagnosis. However, blood glucose levels immediately raised after neratinib discontinuation, although we would have expected a more prolonged effect on glycemia after 18-month neratinib therapy. It is possible that the withdrawal of neratinib together with the add-on dexamethasone therapy (acutely 8 mg twice daily on three subsequent days of chemotherapy every three weeks) has caused the rapid hyperglycemia reoccurrence.

Despite the relatively good tolerability of neratinib, the aggravating side effects of up to grade 3 diarrhea in up to 25% of patients on neratinib combination for cancer therapy currently lowers the motivation for large trials in patients with T2D. However, re-analyses of data from the ExteNET trial concluded that even without antidiarrheal prophylaxis, neratinib-related diarrhea is temporary and not associated with clinical complications (20). Exemplary, the patient in this report has suffered from a short-term grade 1 mild diarrhea, defined by The National Cancer Institute as an increase to <4 stools a day, loss of appetite without changes in eating habits and one episode of vomiting during 24h (21). An anti-sickness regimen could well-control such side effects and the glycemia improvement together with patient’s psychological well-being in response to well-controlled glycemia may well outrange the gastrointestinal side effects.

Of note, the AMPK activator metformin that the patient took as her 1^st^ line diabetes therapy could also have participated in lowering the gastrointestinal side effects of neratinib, as AMPK activation inhibits Cl^-^ cellular transport through inhibition of the cation chloride co-transporter NKCC1 (22) and thus, counteracting the side effect of EGFR inhibition on NKCC1 activation as one mechanism for diarrhea. Metformin could therefore serve as combinatorial therapy together with EGFR/MST1 inhibition not only for diabetes but also to manage side effects mediated by inhibition of EGFR.

Choosing the appropriate therapy for patients with cancer-diabetes co-morbidity is a challenge for both oncologists and diabetologists as potent cancer drugs such as PI3K and AKT inhibitors cause metabolic derangements in patients; hyperglycemia and diabetes develops in up to 50% of patients (23). The PI3K/AKT signaling pathway is a principal regulator of β-cell homeostasis and growth and also an important central hub of insulin signaling in several metabolic organs. Thus, it is no surprise that inhibition of signals in the PI3K/AKT pathway which cause growth retardation in tumors are the same which impair pancreatic β-cell survival and insulin secretion in pancreatic islets and compromise insulin sensitivity and energy homeostasis in the peripheral tissues, collectively worsening glucose metabolism in patients with diabetes. Although there are enormous strategic plans, we are not at a stage yet where cell specific therapy is applied on a routine basis.

Given the risk of diabetes development, there is a high demand of novel drugs targeting only specific components of regeneration/tumorigenic signaling cascades, such as more specific mTORC1 inhibitors (24) as well as the identification of dual targets for the therapy of comorbidities, as suggested by this study.

Without material from biopsy, we are unable to speculate the inhibition of which kinase was responsible for the antihyperglycemic effect in this study; antidiabetic effects have been reported by the inhibition of both EGFR at the level of insulin sensitivity as well as MST1 on the level of β-cell protection. In a previous *in vitro* study, we found that canertinib, an acrylamide-based covalent EGFR inhibitor with a similar structure to neratinib, but lacking MST family activity, did not have any effects on β-cell survival (13, 25). Thus, we currently develop inhibitors with greater selectivity towards MST1 with reduced effects on EGFR to be tested *in vitro* and *in vivo* for their efficacy as well as mechanisms of action of combined versus single MST1/EGFR inhibition for diabetes therapy. Moreover, we cannot fully exclude the steroid effects on glycemia in this report and larger studies are needed to assess the potential role of neratinib on glucose regulation in patients with diabetes.

## Data Availability Statement

The original contributions presented in the study are included in the article/supplementary material. Further inquiries can be directed to the corresponding author.

## Ethics Statement

This study was approved by The Royal Marsden National Health Service Research Ethics Committee (16/SC/0271). The patients/participants provided their written informed consent to participate in this study before registration, which included the publication of all data from the study.

## Author Contributions

Conceptualization, writing, reviewing, and editing: KM and AA. Patient’s examination, data analysis, and original draft: VA. Supervision, study organization, and evaluation: SJ. All authors contributed to the article and approved the submitted version.

## Funding

This work was supported by the German Research Foundation (DFG), JDRF (to AA, KM) and by National Institute for Health Research funding to the Royal Marsden and Institute of Cancer Research Biomedical Research Centre, London, UK.

## Conflict of Interest

KM and AA hold the shared patent WO2016210345A1: Composition and methods for inhibiting mammalian sterile 20-like kinase 1. SJ is consultant for Pfizer, Puma Biotechnology, Eli Lilly, obtained speaker honoraria from AstraZeneca, Pfizer, Eli Lilly and research funding to the institution from AstraZeneca, Puma Biotechnology.

The remaining author declares that the research was conducted in the absence of any commercial or financial relationships that could be construed as a potential conflict of interest.

## Publisher’s Note

All claims expressed in this article are solely those of the authors and do not necessarily represent those of their affiliated organizations, or those of the publisher, the editors and the reviewers. Any product that may be evaluated in this article, or claim that may be made by its manufacturer, is not guaranteed or endorsed by the publisher.
